# Molecular Pathways in Melanomagenesis: What We Learned from Next-Generation Sequencing Approaches

**DOI:** 10.1007/s11912-018-0733-7

**Published:** 2018-09-14

**Authors:** Giuseppe Palmieri, Maria Colombino, Milena Casula, Antonella Manca, Mario Mandalà, Antonio Cossu, for the Italian Melanoma Intergroup (IMI)

**Affiliations:** 1 Unit of Cancer Genetics, National Research Council (CNR), Institute of Biomolecular Chemistry (ICB), Traversa La Crucca 3, Baldinca Li Punti, 07100 Sassari, Italy; 2 PAPA GIOVANNI XXIII Cancer Center Hospital, Bergamo, Italy; 3 Institute of Pathology, Azienda Ospedaliero Universitaria (AOU), Sassari, Italy

**Keywords:** Cutaneous melanoma, Genetics, Melanoma subtypes, Mutation status, Next-generation sequencing, Single-nucleotide (SNV) and copy number (CNV) variations, UV signature

## Abstract

**Purpose of Review:**

Conventional clinico-pathological features in melanoma patients should be integrated with new molecular diagnostic, predictive, and prognostic factors coming from the expanding genomic profiles. Cutaneous melanoma (CM), even differing in biological behavior according to sun-exposure levels on the skin areas where it arises, is molecularly heterogeneous. The next-generation sequencing (NGS) approaches are providing data on mutation landscapes in driver genes that may account for distinct pathogenetic mechanisms and pathways. The purpose was to group and classify all somatic driver mutations observed in the main NGS-based studies.

**Recent Findings:**

Whole exome and whole genome sequencing approaches have provided data on spectrum and distribution of genetic and genomic alterations as well as allowed to discover new cancer genes underlying CM pathogenesis.

**Summary:**

After evaluating the mutational status in a cohort of 686 CM cases from the most representative NGS studies, three molecular CM subtypes were proposed: BRAF^mut^, RAS^mut^, and non-BRAF^mut^/non-RAS^mut^.

## Introduction

Cutaneous melanoma (CM) is one of the most aggressive malignancies and its incidence is continuously increasing in the Caucasian population [1]. Melanoma is characterized by a molecular heterogeneity, considerably greater than that evidenced by the common histopathological features [2]. The melanoma pathogenesis, referred to as melanomagenesis, is based on the acquisition of sequential alterations in specific genes and pathways controlling metabolic or molecular mechanisms and regulating crucial cell functions [3–6]. During past decades, several tumor suppressor genes and/or oncogenes have been reported to be affected by deleterious mutations or structural alterations [3–6].

The introduction of next-generation sequencing (NGS) strategies is speeding up the efforts to identify the whole pattern of mutations involved in the CM pathogenesis [7]. This emphasizes the need to define all or the vast majority of mutational changes in the different melanoma subtypes in order to further progress into the knowledge of the disease onset and to better match subsets of patients to the most appropriate clinical management. In other words, accurate classifications of the spectra of genetic mutations in melanoma tissues may lead to the development of disease-associated biomarkers that help guide the most appropriate clinical management of the different subsets of patients.

In this review, we focus on main molecular features in CM lesions, as assessed by NGS approaches.

## Mutation Status Assessment by NGS-Based Analysis

The NGS assays are used to perform massively parallel sequencing, during which highly redundant fragments of DNA from a single sample are uniformly sequenced [8]. In particular, NGS is a multistep process that typically involves sample acquisition and quality control, DNA isolation and purification, DNA-fragment library preparation, sequencing, and genomic data generation [8]. The most crucial phases in the process are those subsequent to the achievement of the sequence results. Data analysis includes (a) bioinformatics tools for variant identification; (b) variant annotation and prioritization; and (c) interpretation of putative clinical significance. Sequential procedures are used to call somatic sequence variations in tumor tissue samples, after confirming the quality of the sequencing reads [8–10]. Single-nucleotide variations (SNVs), including predicted amino acid changes for non-synonymous mutations and somatic insertion-deletions (indels), are filtered according to the following criteria: (i) coverage of total reads at the variant position; (ii) mutant allele frequency (usually, 10% or more); and (iii) balanced forward and backward reads. Finally, somatic copy number variations (CNVs) are assessed using specific bioinformatics procedures on mapped reads in matched melanoma samples.

Although the NGS methodology may be also used for gene expression studies using RNA substrate, the main application is aimed at identifying mutations and alteration in genes and regulatory elements involved in pathological processes. Prioritizing such genetic variants, by truly distinguishing between “driver” alterations, that are causally related to the cancer development and random “passenger” alterations that have simply accumulated over the tumor growth, represents a major challenge.

## NGS Assays in Cutaneous Melanomas: Overview

Recent years have seen an unprecedented growth in the understanding of genetic and genomic changes in melanoma [11••, 12]. Much of the information comes from NGS analyses, including whole exome (WES) and whole genome (WGS) sequencing approaches, and has been instrumental for the discovery of new genes underlying cancer pathogenesis [13•].

For melanoma, the main WES-based study was performed by the Cancer Genome Atlas on 333 melanomas [14••], while the main WGS-based study by Hayward and colleagues on 183 melanomas [15••]. To focus on CM cases only, we evaluated for mutational status a large cohort of such tumor types excluding the few different melanoma subtypes (acral, mucosal, and ocular) from both previous studies and including data from three additional NGS-based studies: a WES analysis on 147 cases [16], a WES analysis on 135 cases [17], and a WGS analysis on 25 cases [18]. Altogether, 686 CM patients were included into the present study (Table 1).

**Table 1:** Cutaneous melanoma (CM) cases from the NGS-based studies evaluated for the mutational patterns in the present report

Report	Reference no.	Total cases into the study	CM cases included into the present analysis
The Cancer Genome Atlas (TGCA)	[14••]	333	317
Hayward et al.	[15••]	183	140
Krauthammer et al.	[16]	147	109
Hodis et al.	[17]	121	95
Berger et al.	[18]	25	25
Total cases		809	686

NGS-based findings confirmed the role of more well-known genes (BRAF, NRAS, TP53, CDKN2A, PTEN, MAP 2K1-2, KIT, and RB1) and recently identified genes (NF1, ARID2, PPP6C, RAC1, DDX3X, and IDH1) in melanoma. The scenario of genetic alterations contributing to melanoma gene signature was completed by somatic CNVs, such as gene amplifications in CCND1, CDK4, KIT, MITF, and TERT as well as gene deletions in CDKN2A and PTEN. However, additional candidate cancer genes are likely to be identified since criteria currently applied in NGS analyses for selecting significant mutations do not consent to achieve full statistical power, and driver genes with a mutation frequency ≤ 2% can be missed [19]. Furthermore, different types of mutations are yet to be investigated in detail. In particular, mutations at the 5′-UTR or promoter regions, for their functional impact on gene expression levels, and within the 3′-UTR, for possible interference with the transcript translation activity [20], deserve a more in-depth examination as it is probable that variants at these levels may currently be under-represented. Nonetheless, mutations in other non-coding regions—such as those in transcription factor binding sites [21] or in splicing regions with generation of new transcript isoforms [22]—also deserve a more accurate classification. Finally, the functional role of synonymous mutations as well as of fusion events and all structural changes in melanomagenesis is still to be fully understood.

## NGS-Based Mutation Analysis: Results

Considering data from all five studies on CMs, about 75% of genomic sequence variations are represented by C > T substitutions, with another small fraction (< 5%) constituted by CC > TT transitions. These variants are due to the mutagenic effects of ultraviolet (UV) radiation on exposed skin and the entire set of these are indicated as the UV mutation signature [23–24]. For the same UV effects on mutagenesis and interventions of genetic factors [25], the genomic DNA from CM samples has one of the highest mutational burden as compared to that from other cancer types (contributing to the increased responsiveness to immunotherapy [26]). As a confirmation of this, non-cutaneous (i.e., ocular and mucosal) melanomas present a markedly lower mutational load and lack the UV signature [15^••^, 27]. Overall, the mutation rate in melanomas occurring on chronically sun-exposed skin was found to be approximately five times higher than those on the skin not subject to sun damage, confirming previously reported data (ratio of about 21 mutations/Mb vs. 5 mutations/Mb in the two subgroups, respectively [28•]).

In Table 2, all deleterious gene mutations in our series of 686 melanomas from the above indicated five studies [14••, 15••, 16–18] are reported. Overall, it was confirmed that the most frequent somatic alterations are represented by mutations in BRAF (340; 49.6%) and RAS (202; 29.4%—with NRAS isoform involved in about 94% of RAS-mutated cases). Therefore, about three fourths (76.6%) of CM cases presented a pathogenetic mutation in these two oncogenes. The occurrence of oncogenic RAS mutations is reported in 8/340 (2.4%) cases carrying oncogenic BRAF mutations, confirming that coexistence in the same melanomas of pathogenetic mutations in these two genes is very infrequent. In an additional but still limited fraction of cases (less than 5%), co-occurring *BRAF* and *RAS* mutations harbored either a non-activating *BRAF* variant together with an oncogenic *RAS* mutation or a *RAS* variant not recognized as oncogenic together with an activating *BRAF* mutation. According to the mutation prevalence, the remaining melanoma driver genes could be divided into three groups: one (TP53, NF1, CDKN2A, and ARID2), with mutation frequency between 10 and 20%; the second (PTEN, PPP6C, RAC1, and DDX3X), with mutation frequency between 5 and 9%; and the third one (16 genes), with mutation frequency < 5% (Table 2). Finally, less than one tenth of CMs (45/686; 6.6%) was negative for any genetic alteration.

**Table 2:**
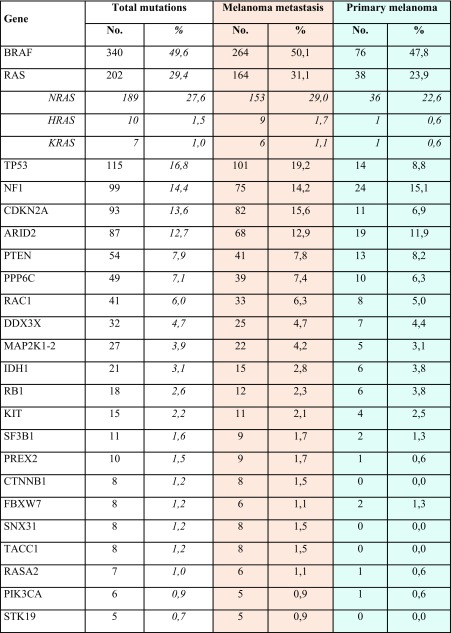

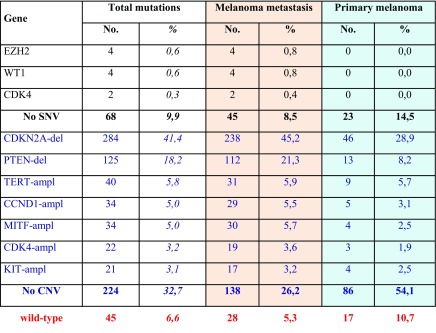
Distribution of genetic and genomic alterations among the 686 CM cases. SNVs, in dark; CNVs, in blue; del, gene deletion; ampl, gene amplification. In red, all cases negative for any genetic/genomic alteration (fully wild-type)

**Fig. 1:**
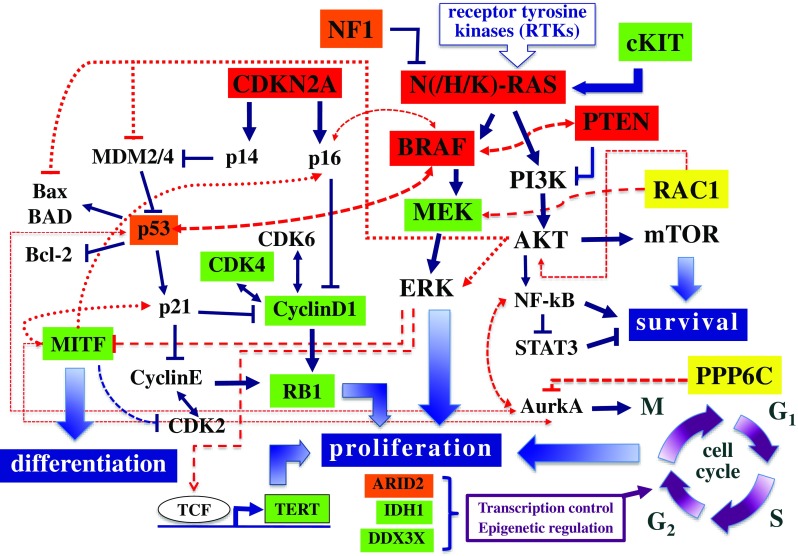
Signal transduction pathways involved in melanomagenesis. Genes are evidenced according to the prevalence of alterations, including SNVs and CNVs, in the series of 686 CM samples: in red, ≥ 20% of cases; in orange, ≥ 10 to < 20%; in yellow, ≥ 5 to < 10%; in green, ≥ 2 to < 5%

Considering the CNVs whose functional role has been previously demonstrated (see above), inactivation of CDKN2A and PTEN tumor suppressor genes was confirmed to be frequently implicated in melanomagenesis (Table 2). Overall, about one third of CMs (224/686; 32.7%) lack any structural rearrangement in the candidate genes. In Fig. 1, all main genes and related pathways characterized by the NGS-based analyses are represented, evidencing the frequencies of the identified molecular alterations (summing SNVs and CNVs).

**Fig. 2:**
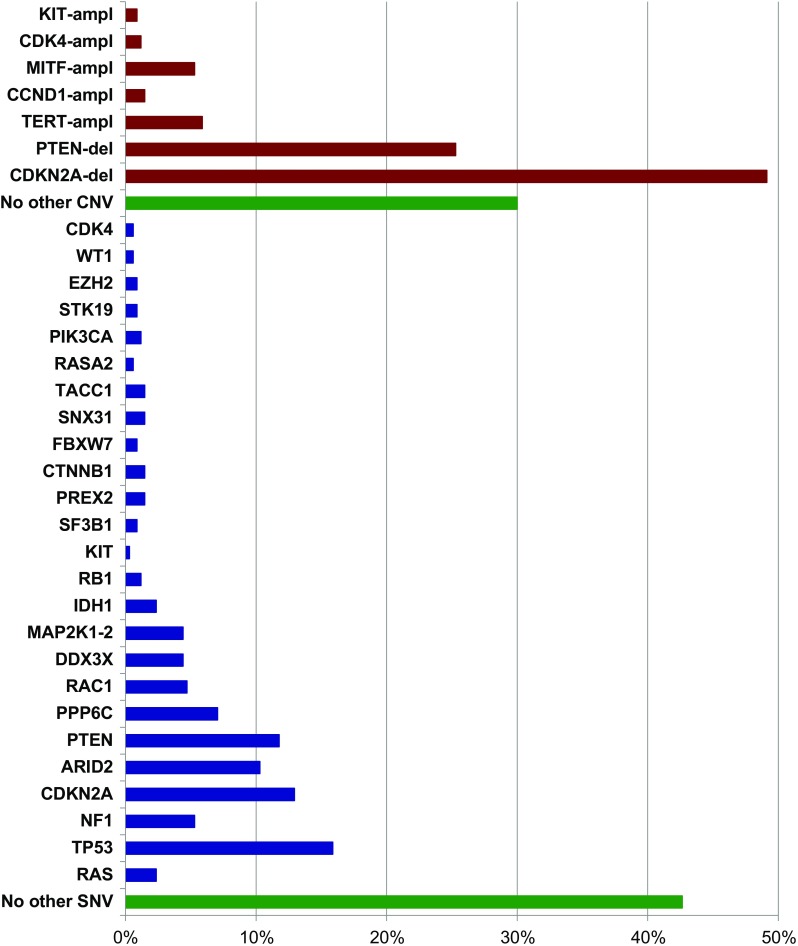
Gene mutation frequencies in the 340 cases with the BRAF^mut^ subtype. In red, copy number variations; in blue, pathogenetic mutations; in green, lack of additional genetic alterations. ampl, amplification; del, deletion

**Fig. 3:**
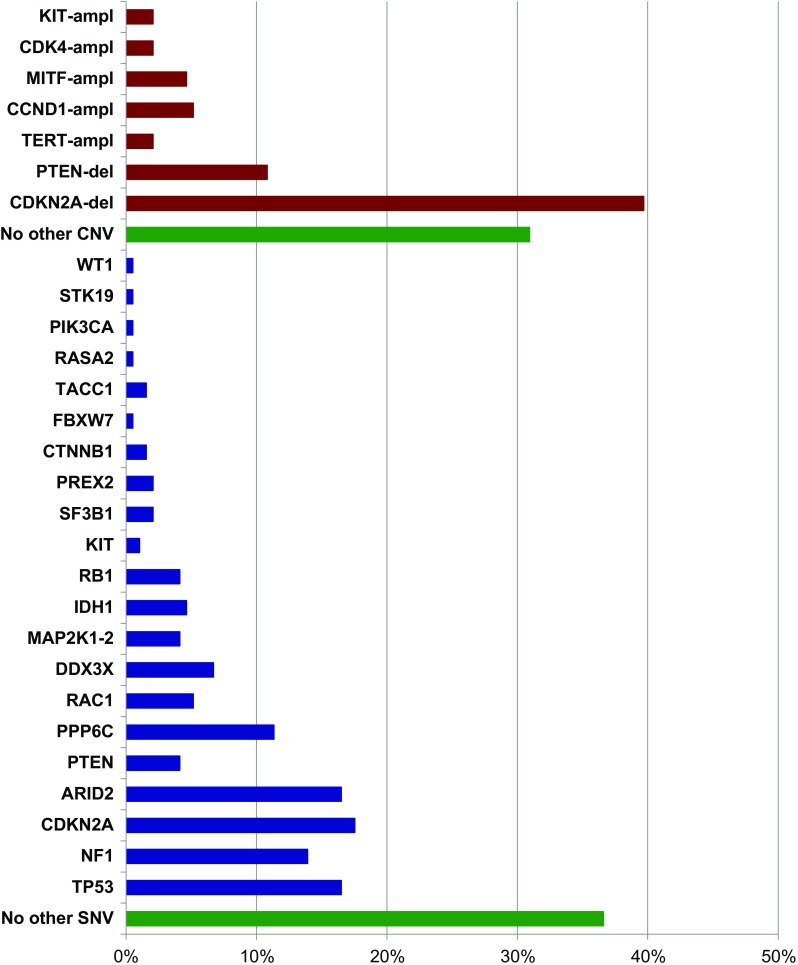
Gene mutation frequencies in the 194 cases with the RAS^mut^ subtype. In red, copy number variations; in blue, pathogenetic mutations; in green, lack of additional genetic alterations. ampl, amplification; del, deletion

**Fig. 4:**
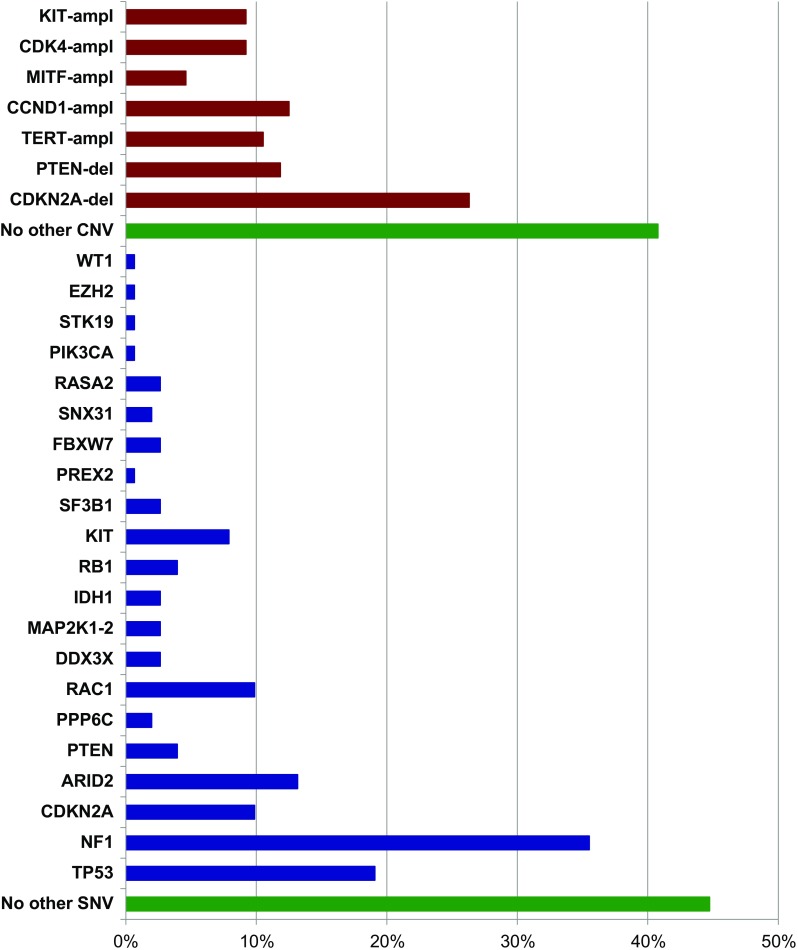
Gene mutation frequencies in the 152 cases with the non-BRAF^mut^/non-RAS^mut^ subtype. In red, copy number variations; in blue, pathogenetic mutations; in green, lack of genetic alterations. ampl, amplification; del, deletion

The Cancer Genome Atlas classified melanomas in four molecular subtypes: BRAF, RAS, and NF1 mutation carriers, along with the so-called triple wild-type (lack of mutations in all three genes) [14••]. However, NF1 mutations were found—albeit at a lower frequency—in the BRAF^mut^ and RAS^mut^ subgroups also, not allowing to appropriately define the subset of NF1^mut^ melanomas as a real independent molecular subtype. This was further confirmed in our series of NGS studies and, therefore, the following three molecular subtypes are here proposed: BRAF^mut^ (Fig. 2), RAS^mut^ (Fig. 3), and non-BRAF^mut^/non-RAS^mut^ (Fig. 4). From the evaluation of the mutational status, it appears clearly evident that, even in subtypes characterized by specific main mutations (BRAF^mut^ and RAS^mut^), many additional genes are recurrently mutated (Figs. 2, 3, and 4). For these reasons, there is an increasing need of introducing panel-based testing approaches in order to provide an opportunity for patients to achieve a deeper look at their molecular heterogeneity through an improved clarification of their mutational status [29–32]. Such approaches pave the way for the development of tailored patients’ testing and clinical management.

A description of the genetic alterations identified by NGS-based analyses and characterizing the different molecular subtypes in our series of CM is here provided.

## BRAF/MAP 2K1-2 Genes

The RAF kinase family consists of three proteins (ARAF, BRAF, and CRAF), which are all part of the signal transduction cascade named *mitogen-activated protein kinase* (MAPK) pathway and whose kinase activity is physiologically induced by activation of the upstream RAS protein [33]. Activated BRAF promotes activity of the MEK1-2 kinases, which in turn activate ERK1-2 as final effectors of the MAPK pathway (Fig. 1). In our series, BRAF is mutated in about half (340/686; 49.6%) of cases (Table 2). The most prevalent mutation (about 90% of cases) is represented by a substitution of a valine with glutamic acid at codon 600 (BRAF^V600E^). Few additional BRAF variants at codon 600 (BRAF^V600^ mutations) or, less frequently, in other codons within the BRAF kinase region (in particular, BRAF^K601E^) have been demonstrated to activate the MEK1-2 downstream pathway effectors [3, 11••, 12, 14••]. All these BRAF mutations promote a constitutive stimulation of cell proliferation and tumor growth. Since BRAF is also mutated in common nevi, its oncogenic activation is supposed to be a necessary but not sufficient condition for melanoma development (being considered as an initiation event in melanocyte transformation) [34]. In our CM cohort, BRAF^mut^ carriers were characterized by a higher frequency of PTEN and CDKN2A alterations (including both gene mutations and deletions) in comparison to the RAS^mut^ and non-BRAF^mut^/non-RAS^mut^ subtypes (Table 3). As a confirmation of this, it was recently demonstrated that activation of MEK in BRAF^mut^ cells promotes the development, growth, and maintenance of melanoma in vivo when PTEN and/or CDKN2A losses coexist [35].

**Table 3:**
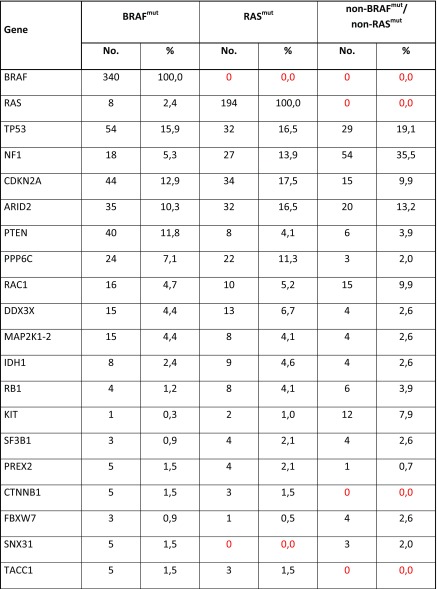

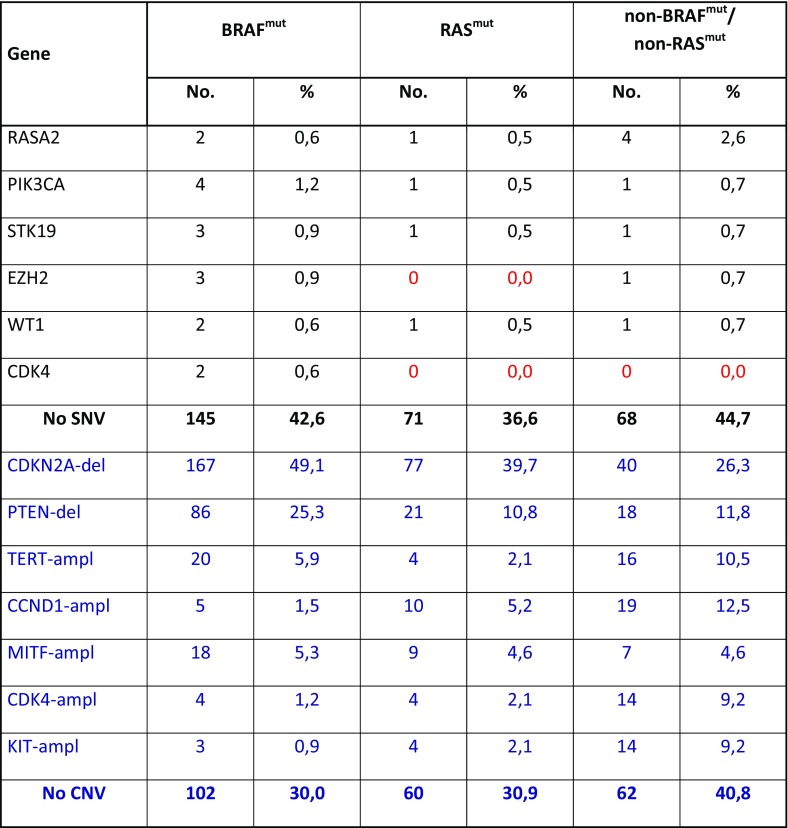
Distribution of genetic and genomic alterations among the three molecular subtypes of CM cases. SNVs, in dark; CNVs, in blue; del, gene deletion; ampl, gene amplification. In red, negative cases

Finally, BRAF mutations typically occur in younger patients [14••], are less associated with the UV mutation signature, and are more prevalent in melanomas arising on not chronically sun-exposed skin areas [12, 36, 37]. Although the prognostic significance for BRAF mutants remains unclear since controversial results were reported [38–41], the occurrence of BRAF mutations seems to have no impact on the disease-free interval from diagnosis of first-ever melanoma to first distant metastasis, but may worsen the overall survival after disease progression [42•].

MEK1 and MEK2 represent targets downstream of the RAS-RAF-MAPK cascade (Fig. 1). In our series, a small fraction of cases (27/686; 3.9%) carry mutations in the corresponding genes encoding these two kinases (MAP 2K1 and MAP 2K2, respectively). Activating mutations in MAP 2K1-2 represent one of the multiple mechanisms of resistance to BRAF and MEK inhibitors. No difference in the distribution of MAP 2K1-2 mutations among the three CM subtypes was found.

## RAS/NF1/RAC1/PREX2 Genes

The three tissue-specific isoforms—HRAS, KRAS, and NRAS—of the RAS gene family encode small GTPase proteins bound to the cytoplasmic membrane. Among them, NRAS is the most commonly mutated gene in CMs (189/686, 27.6%), whereas the involvement of the other two isoforms is minimal (HRAS, 1.5%; KRAS, 1.0%) (Table 2). The oncogenic RAS is able to activate downstream cytoplasmic proteins [11••, 12]: RAF and phosphatidylinositol 3-kinase (PI3K) (Fig. 1). Similarly to BRAF, NRAS mutations occur almost exclusively in a single gene codon (Q61, about 90% of cases); in the remaining 10% of cases, the mutated codon is G12 or G13. Unlike BRAF, NRAS mutations have been described at similar frequencies in melanomas arising in different skin areas [12, 37].

NF1 is a tumor suppressor gene encoding a negative regulator of RAS [43, 44]. NF1 was found mutated in about 14% of CMs from our cohort, partially associated with other mutations in RAS-MAPK pathway genes (such as RASA2, a RASopathy gene activating the RAS-dependent kinase effectors; [45]). Melanomas carrying NF1 mutations present with a higher mutational burden and a markedly evident UV mutation signature [44]. Loss of NF1 function has been related to increased resistance to BRAF and MEK inhibitors [46, 47]. In our series, NF1 mutations have the lowest rate (18/340; 5.3%) in BRAF^mut^ subtype, an intermediate rate (27/194; 13.9%) in RAS^mut^ subtype, and the highest prevalence (54/152; 35.5%) in the non-BRAF^mut^/non-RAS^mut^ subtype (Table 3).

NGS analyses revealed pathogenetic mutants in the RAC1 gene, encoding a GTPase able to induce the activity of RAS-dependent pathways and stimulate cell proliferation and migration [16, 45, 48]. Activation of RAC1 may be promoted by mutated PREX2 [18, 48–50]. This gene encodes a PTEN-binding protein and is mutated in 1.5% of CMs from our series (Table 2). In immunodeficient mice, the mutated PREX2 was found to enhance tumorigenesis, suggesting to play an oncogenic role [49].

## CDKN2A/RB1/CDK4/CCND1 Genes

The interaction between cyclin D1 (CCND1) and the serine/threonine kinase CDK4/6 is strongly regulating cell proliferation. Once activated, the CDK4/6 protein interacts with the RB1 protein in inducing transcriptional events necessary for cell cycle progression from G1 to S phase [51, 52]. This process is negatively regulated by p16^CDKN2A^ and alterations inactivating this tumor suppressor gene are thought to be related to disease progression: absent in nevi, occurring in primary tumors, and steadily increasing in metastatic melanomas [3, 11••, 12]. The inactivation of p16^CDKN2A^ is due to either genetic (mutations, deletions) or epigenetic (promoter methylation) mechanisms, whereas the amplification of CCND1 is particularly frequent in melanomas negative for BRAF/NRAS mutations, in chronically sun-exposed skin areas [53]. In our series, CDKN2A was found to carry gene mutations in 13.6% of cases (mostly, in RAS^mut^ subtype) and gene deletions in 41.4% of cases (mostly, in BRAF^mut^ subtype) (Table 3). The CCND1 gene amplification was found in 5.0% of the cases, with the highest prevalence (12.5%) in the non-BRAF^mut^/non-RAS^mut^ subtype, as compared to BRAF^mut^ (1.5%) or RAS^mut^ (5.2%) ones (Table 3).

## PTEN/PI3K/AKT/mTOR/KIT Genes

The PI3K-dependent pathway, including the signal transduction cascade with PTEN, AKT, and mTOR proteins, is a strong regulator of melanoma growth and survival [53–55]. In physiological conditions, activated PIK3CA—mostly, consequence of RAS activation—increases the intracellular level of PIP2/PIP3 phosphoinositoles (which are instead reduced by PTEN activity) and the downstream substrates AKT and mTOR are in turn activated (Fig. 1). In our series, constitutive activation of this pathway is due to the acquisition of PTEN alterations (gene mutations and allelic deletions: 7.9 and 18.2%, respectively), with very few cases (0.9%) mutated in PIK3CA gene (Table 3). Oncogenically activated BRAF and silenced PTEN cooperate in melanoma formation and progression, resistance to BRAF/MEK inhibitors, and interference with tumor immune infiltration (suggesting that PI3K-pathway inhibition may represent a strategy in patients receiving targeted therapy in combination with immune checkpoint inhibitors) [56–59].

The mutated or amplified KIT gene, encoding a tyrosine kinase receptor of the cell membrane [53], may activate both MAPK and PI3K-AKT downstream pathways (Fig. 1). In our series of CMs, 2.2% (15/686) of the cases carried a KIT mutation, with a markedly lower prevalence in the BRAF^mut^ and RAS^mut^ subtypes (0.3 and 1.0%, respectively), as compared to that (7.9%) found in the non-BRAF^mut^/non-RAS^mut^ subtype (Table 3). Again, coexistence of pathogenetic mutations in BRAF or NRAS or KIT genes is rare, further confirming that oncogenic activations of such genes are mutually exclusive. Analogously, KIT gene amplifications were observed at the low frequency (3.1%); they were more prevalent in non-BRAF^mut^/non-RAS^mut^ subtype (9.2%) than in the other subtypes (BRAF^mut^, 0.9%, RAS^mut^, 2.1%) (Table 3).

## TP53 Gene

Inactivation of the TP53 tumor suppressor gene plays an important role in CM pathogenesis [11••, 12, 36]. Normal intracellular levels of the p53 protein promote the control of cell cycle progression, whereas TP53 upregulation induces mechanisms of protective apoptosis. For its capability in responding to genotoxic damages, p53 levels have been implicated in enhancing or contrasting the UV-driven melanomagenesis [60, 61]. In melanoma, a reduction of the p53 protein levels is commonly reported [3, 11••, 12]. The downregulation of TP53 gene may be due to several mechanisms [62–64]: inactivation of p14^CDKN2A^ causing MDM2 overexpression, or increased stability of MDM2-4 proteins induced by activation of the receptor tyrosine kinase AXL, or TP53 gene silencing through genetic changes (mostly, mutations) or epigenetic deregulations (Fig. 1). As confirmation of previously reported interactions between activated BRAF and silenced TP53 [3, 11••, 12], restoring the intracellular p53 levels may sensitize BRAF^mut^ melanomas to BRAF inhibitors [65].

In our series, pathogenetic mutations of TP53 were present in 16.8% of cases, with a quite similar distribution in the BRAF^mut^ (15.9%), RAS^mut^ (16.5%), and non-BRAF^mut^/non-RAS^mut^ (19.1%) subtypes (Table 3).

## PPP6C/ARID2/IDH1 Genes

The NGS-based mutation analyses revealed a high frequency of mutations in the genes encoding proteins which regulate the epigenetic mechanisms of gene transcription and expression [66, 67].

Mutations of the PPP6C gene impair the activity of the Aurora A kinase (AurkA), one of the main regulators of the M phase progression into the cell cycle (Fig. 1), causing chromosome instability [68]. Recently, a tight interaction between PPP6C-AurkA and MAPK pathways in controlling proliferative activity and apoptosis in melanoma cells has been demonstrated, contributing to increase the levels of tumor heterogeneity and conferring a resistance signature in BRAF- and NRAS-mutated melanomas [69, 70]. The PPP6C protein deficiency is able to induce cutaneous tumorigenesis in mice treated with the DMBA carcinogen, strongly suggesting a role as tumor suppressor gene [71]. Finally, PP6C mutations were associated with increased autophagy in vitro and in vivo melanoma samples [72]. The PPP6C gene is mutated in 7.1% of CM cells, with similar prevalence in the different stages of tumor progression (6.3% in primary and 7.4% in metastatic melanoma; Table 3).

Among the genes involved in epigenetic regulation, ARID2 encodes subunits of a tumor suppressor complex involved in chromatin remodeling, which has been implicated in pathogenesis of several cancers [36, 73, 74]. Inactivation of genes able to regulate the chromatin remodeling, including ARID2, may enhance the infiltrate of cytotoxic T cells and sensitize melanoma cells to their killing activity [75•]. Furthermore, IDH1 acts as the main source of cytosolic NADPH, protecting cells against reactive oxygen species (ROS) and radiations [76, 77]. Inactivating mutations of these two genes occur in 12.7% (ARID2) and 3.1% (IDH1) of CMs in our series, independently on the BRAF/RAS mutational status (Table 3).

## Other Genes in Melanomagenesis

DEAD/H (Asp-Glu-Ala-Asp/His) polypeptide 3,X-linked (DDX3X) is a gene involved in the transcription control by regulating RNA homeostasis [14••, 15••]. DDX3X is involved in the epigenetic regulation of gene expression and its activation induces resistance to kinase inhibitors [78]. The DDX3X mutations were found in 4.7% of CMs, mostly associated with BRAF or RAS mutations (Table 3). Among genes epigenetically modulated by DDX3X, CTNNB1 encodes the beta-catenin protein and is mutated in 1.2% of CMs (Table 3). The CTNNB1 mutations were exclusively observed in the BRAF^mut^ and RAS^mut^ subtypes, confirming the functional interaction between beta-catenin signaling and MAPK pathway activity (also through the involvement of recently described p21-activated kinases) [79, 80]. Recent evidence revealed that beta-catenin signaling may be related to a hypo-methylation status, a reduced T cell infiltration, an immune escape condition, and a lower immunogenicity [81, 82].

## TERT

The telomerase reverse transcriptase gene (TERT) maintains telomere homeostasis, counteracting the progressive shortening of telomeres during mitoses [83]. Telomerase is present in its active form in stem cells, while it is inactivated in most somatic cells. In majority of cancers, the reactivation of telomerase is crucial for tumor cell survival. Mutations in the TERT gene promoter usually coexist with MAPK pathway activation [84]. TERT was found amplified in 5.8% of CMs from our cohort, with no particular association with the BRAF/NRAS mutational status (Table 3).

## MITF

MITF is acting downstream of the BRAF-MEK-ERK signal transduction pathway (Fig. 1). In addition to its involvement in skin pigmentation, MITF plays an important role in proliferation and differentiation of melanocytes [11••, 12]. However, the mechanism of action of MITF is rather complex: low or absent levels of this protein predispose cells to apoptosis; intermediate MITF expression levels promote cell proliferation and survival; high concentration of intracellular MITF proteins induces antiproliferative effects [85]. An association was reported between MITF overexpression, partially regulated by AurkA, and MAPK pathway activation in controlling melanoma cell proliferation and migration [86]. MITF amplification was found in 5.0% of CMs, regardless of the BRAF/RAS mutational status (Table 3).

## Mutation Distribution Between Primary and Metastatic Cutaneous Melanomas

Our series of 686 skin melanomas consists of 159 (23.2%) primary tumors and 527 (76.8%) metastatic melanomas. As shown in Table 2, some genetic alterations (BRAF, NF1, ARID2, PTEN, PPP6C, RAC1, DDX3X, MAP 2K1-2, IDH1, RB1, KIT, SF3B1, FBXW7, and PIK3CA mutations; TERT and KIT amplifications) present similar frequencies between primary and metastatic CMs. Alterations may represent initial events promoting cell proliferation and survival, but additional molecular alterations are needed for melanocytic transformation. Prevalence of other genetic alterations (RAS, TP53, CDKN2A, PREX2, and RASA2 mutations; CDKN2A and PTEN deletions, CCND1, MITF, and CDK4 amplifications) is higher in metastatic that in primary melanomas, most likely due to the expansion of cell subpopulations during tumor progression. Finally, a few genetic alterations (CTNNB1, SNX31, TACC1, STK19, EZH2, WT1, and CDK4 mutations) are present in metastatic subsets only, suggesting that these play a role in the advanced phases of the disease.

Overall, the fractions of cases negative for any pathogenetic SNV (14.5 vs. 8.5%) or CNV (54.1 vs. 26.2%) were markedly higher in primary melanomas than in metastatic lesions, respectively.

## Conclusions

In addition to the genetic variations, data are emerging that support a role of epigenetic modifications in melanoma pathogenesis [66, 67]. Epigenetic changes having an impact on the expression levels of candidate genes are represented by:

- post-translational variations and chromatin remodeling;
- methylation of cytosine-guanine dinucleotides (CpG);
- gene silencing performed by non-coding RNA.

Small (sncRNAs; < 200 nucleotides) and long (lncRNAs; > 200 nucleotides) non-coding RNAs as well as microRNAs (miRNAs; approximately, 22 nucleotides long) can regulate gene expression through transcription modifications and mRNA stability interfering with translation. As example, a copy number analysis indicated the pathogenetic coexistence of MITF amplification and expression of a specific lncRNA (SAMMSON) in a subset of melanomas. Aberrant DNA methylation [87, 88], up-/downregulation of miRNAs’ activity in controlling key signaling pathways responsible for the melanoma cell growth [89], and occurrence of synonymous mutations with a functional role [90] could further affect melanomagenesis and/or disease behavior.

Considering the entire scenario, the paradigm of melanoma pathogenesis seems to be highly more complicated than expected. A more in-depth knowledge of genes and molecular pathways might help in discriminating melanoma patients for response/resistance to specific treatments (predictive factors) or clinical outcome (prognostic factors). Molecular classification will increasingly be an integral part of the management of patients with cutaneous melanoma.
